# Impaired social concept processing in persons with autistic-like traits

**DOI:** 10.1038/s41598-023-42889-2

**Published:** 2023-09-21

**Authors:** Agustina Birba, Joana López-Pigüi, Inmaculada León Santana, Adolfo M. García

**Affiliations:** 1 Cognitive Neuroscience Center, University of San Andrés, Vito Dumas 284, B1644BID Victoria, Buenos Aires Argentina; 2 Instituto Universitario de Neurociencia, Universidad de La Laguna, Tenerife, Spain; 3 Department of Psychology, Faculty of Health Sciences, University of Hull, Kingston Upon Hull, UK; 4 Facultad de Psicología, Universidad de La Laguna, Tenerife, Spain; 5 Global Brain Health Institute, University of California San Francisco, San Francisco, USA; 6 Departamento de Lingüística y Literatura, Facultad de Humanidades, Universidad de Santiago de Chile, Santiago, Chile

**Keywords:** Language, Social neuroscience

## Abstract

Situated models suggest that social concepts are grounded in interpersonal experience. However, few studies have tested this notion experimentally, and none has targeted individuals with reduced social interaction. Here, we assessed comprehension of text-level social and non-social concepts in persons with and without autistic-like traits. Participants read a social and a non-social text and answered questionnaires targeting social and non-social concepts, respectively. We compared behavioral outcomes, gauged their contribution to subject-level classification, and examined their association with validated measures of autism. Persons with autistic-like traits showed selective deficits in grasping text-level social concepts, even adjusting for intelligence, memory, and vocabulary. Also, social concept comprehension was the only variable that significantly classified between groups. Finally, social concept outcomes correlated negatively with measures of autism, including social interaction. Our results suggest that reduced interpersonal experience selectively compromises text-level social concept processing, offering empirical constraints for situated models of social semantics.

## Introduction

Social concepts (complex categories evoking interpersonal behaviors, traits, or events) are a hot topic in semantic memory research. Situated accounts underscore their grounding in interpersonal experience, which would provide contextual anchorage for their understanding and processing. Yet, few studies have tackled this notion experimentally and none has tested the key hypothesis that social concept processing should be distinctly undermined in persons with reduced social interaction. To bridge this gap with naturalistic materials, we examined comprehension of a social text (ST, rich in social concepts) and a non-social text (nST, devoid of social concepts) in individuals with and without autistic-like traits, accounting for relevant factors and examining correlations with measures of autism, including social interaction.

Social concepts capture salient aspects of interpersonal scenarios, such as person-specific knowledge, other-directed behaviors, and pro/anti-social traits^1–4^. Linguistically, these are manifested by positively or negatively valenced words that evoke socialness, including verbs (e.g., *help*, *resent*), nouns (e.g., *kindness*, *envy*), and adjectives (e.g., *friendly*, *jealous*)^1^. Despite overlaps with other abstract categories^5^, socially-laden words comprise a distinguishable semantic cluster within the lexicon^6^ and distinctly engage brain regions subserving theory of mind and other socio-cognitive domains^2, 4, 7–9^. Moreover, social content represents a latent factor accounting for inter-individual neural commonalities during activation of abstract concept features^10^. Accordingly, social concepts arguably constitute a distinct category amidst other forms of semantic knowledge^1, 2, 4^.

Given their scope, social concepts typically involve perceptually heterogeneous exemplars, highly indeterminate referents, and diverse thematic associations^5^. Thus, unlike other categories rooted in consistent sensorimotor experiences (e.g., body-action concepts), they are likely grounded through continued interpersonal exchanges and socially shared linguistic labels^5, 11^. While sustained contact with others may be important to establish diverse abstract categories^5, 12^, this requisite proves particularly critical for social concepts. Indeed, as detailed in situated accounts of cognition, concepts are grasped through first-hand experience with the scenarios in which they occur and to which they allude^13–15^, meaning that social concepts crucially hinge on actual social immersion. Their understanding, indeed, would imply reactivating multiple memory traces of such lived events^13, 16^, as implied in neuroimaging studies showing that social cognition regions are activated during social word processing in semantic decision^7^, verb-noun association^17^, and sentence comprehension^18–20^ tasks. Accordingly, just like our grasping of social concepts shapes interpersonal experiences^1^, so, too, these experiences would shape our grasping of social concepts.

Previous studies show that socio-cognitive domains, such as moral judgment, emotional dysregulation, and theory of mind, are distinctly affected in antisocial^21^ and lonely^22^ individuals, including victims of bullying^23^. More particularly, patients with altered socio-interactive conduct exhibit selective social concept deficits, which correlate with anatomo-functional alterations along social cognition brain networks^9, 24^. Accordingly, social concept processing skills might be related to the richness of social experience. However, the situated account of social concepts has not yet been tested against a critical model: persons with and without autistic-like traits—defined by the gold-standard Autism Spectrum Quotient (AQ)^25^ as high AQ and low AQ, respectively.

In addition to introversion, depression, and low conscientiousness^26^, high AQ individuals are typified by limited interpersonal exchanges, poor reciprocal social interaction and dialogue, and a preference for isolated activities^26, 27^. Reduced interpersonal communication is, in fact, one of their predominant characteristics^26, 27^. These features are almost identical in people with an actual diagnosis of autism spectrum disorder (ASD)^25^. Individuals with high AQ score lower than those with low AQ on social cognition measures^28–30^, and their performance correlates with scores in the Autism Diagnostic Observation Schedule-2 (ADOS-2)—the gold-standard instrument in ASD research, including subscales of reciprocal social interaction and interpersonal communication skills^31^. Interestingly, some such impairments seem uninfluenced by broader cognitive skills, including general intelligence, working memory, and vocabulary^32, 33^. Therefore, high AQ scorers offer a critical model to test whether social concept processing is related to interpersonal experience, and whether this link is mediated by more general cognitive traits.

Importantly, this issue can be studied with ecological validity through naturalistic texts. Most social concept research^17, 34, 35^ has employed single-item stimuli, overlooking the contextual anchorage needed to capture words’ precise social implications. For instance, while the verb *promise* may or may not entail a social commitment when presented in isolation, it acquires rich interpersonal significance in the sentence *She promised she would always be there for him*. Indeed, textual context prompts specific emotional, empathic, and mentalistic operations that ground these concepts in actual social experience^2, 9, 24^. Though blind to the individual role of such socio-cognitive variables, text-level paradigms thus enable more naturalistic assessments of social concepts, addressing calls for ecologically valid insights on the construct^9, 24^ and on language processing at large^36–42^.

Against this background, we employed a naturalistic text paradigm assessing social and non-social concept comprehension via multiple-choice questionnaires^24^. Importantly, this task has revealed selective social concept deficits in other populations with socio-interactive atypicalities^24^. We established (sub-clinical) autistic traits via the AQ. Also, to better capture the social profiles of high AQ participants, we administered module 4 of the ADOS-2 scale, computing its total score and outcomes in relevant subscales. Based on previous findings, we predicted that high AQ participants would be outperformed by low AQ persons on the ST (but not on the nST) questionnaire, irrespective of intelligence, working memory, and vocabulary skills. Second, we anticipated that ST outcomes would robustly classify between low AQ and high AQ participants at the individual level. Finally, we predicted that the greater the social detachment of high AQ participants, the lower their capacity to grasp ST information. With this approach, we aim to shed new light on the role of situated interactive experiences in grounding social concepts.

## Methods

### Participants

Participants were drawn from a large pre-screening group of 878 students. All of them voluntarily completed an online version of the AQ, yielding a mean total AQ score of 18.24 (*SD* = 5.5). These individuals were contacted through various channels, including the Canarian Association for Autism Spectrum Disorder, online platforms from faculties at Universidad de La Laguna (Spain), and a student-support program from the same university. Recruitment efforts also included oral invitations to students during lectures. Participants were considered to have a high AQ if their scores were greater than 30 (2 *SD*s above the overall group’s mean), and to have low AQ group if their scores fell between the mean and one *SD* below it (keeping within the range of 13–18 to avoid extremely low values, as in previous research)^28^.

**Figure 1:**
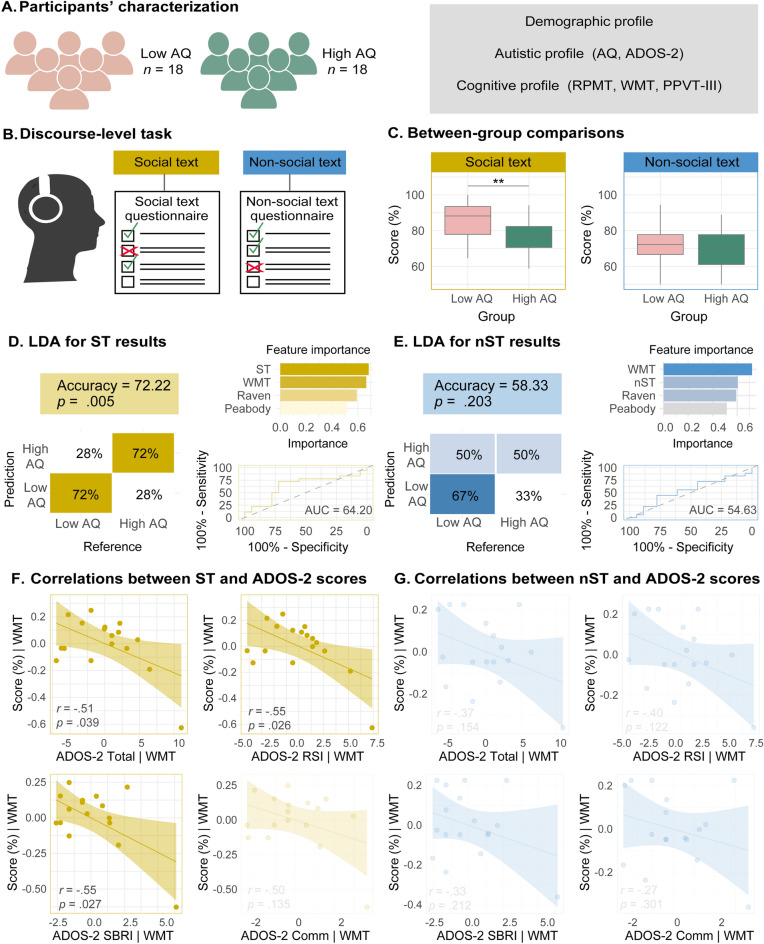
Experimental setup and results of the within-text analyses. (**A**) Participants were characterized in terms of their demographic, autistic, and cognitive profiles. (**B**) In the discourse-level task, participants listened to an ST and an nST, each text being followed by 16 comprehension questions. The order the texts was counterbalanced across participants. (**C**) Between-group comparisons revealed significantly lower scores for the high AQ than for the low AQ group on the ST, but not on the nST. (**D**,**E**) Classification results for the ST (panel **F**) and the nST (panel **G**), together with RPMT, WMT, and PPVT-III scores, depicted with a confusion matrix (left inset), feature importance rankings (top right inset), and a ROC curve (bottom right inset). (**F**) Pearson’s partial correlations, covaried by WMT scores, showed that ST outcomes were negatively associated with the ADOS-2 total score (top left), the ‘reciprocal social interaction’ subscore (top right), and the ‘stereotyped behaviors and restricted interests’ subscore (bottom left), but not the ‘communication subscore’ (bottom right). (**G**) Pearson’s partial correlations revealed non-significant associations between nST performance and any each of the ADOS-2 measures, covaried by WMT scores. *ADOS-2* autism diagnostic observation schedule-2, *low AQ* low autism spectrum quotient, *high AQ* high autism spectrum quotient, *RSI* reciprocal social interaction, *SBRI* stereotyped behaviors and restricted interests, *Comm* communication, *WMT* working memory task, *ST* social text, *nST* non-social text, *PPVT-III* Peabody Picture Vocabulary Test, *RPMT* Raven’s progressive matrices test-III. Double asterisks (**) indicate significant differences after covariation for RPMT, WMT, and PPVT-III outcomes.

The final sample comprised 36 native Spanish speakers, 18 with high AQ and 18 with low AQ (Fig. 1A). This sample size reaches a power of 0.88 (Supplementary material, Power estimation section). All participants were right-handed, had normal or corrected-to-normal vision and hearing, and presented no history of psychiatric disorders, neurological diseases, primary language deficits or substance abuse. Both groups were matched for sex, age, and years of education.

All participants were assessed for non-verbal intelligence, via the Raven’s Progressive Matrices Test [RPMT^43^]; mnesic skills, through a verbal working memory task [WMT^44^]; and linguistic skills, via the Peabody Picture Vocabulary Test III [PPVT-III^45^]—for a description of these tests, see Supplementary material, Cognitive assessment. All assessments were conducted in person by one of the researchers (JLP) in a quiet room with dim lighting. Participants in the high AQ group were also evaluated via module 4 of the ADOS-2 scale. In all cases, participants first completed a demographic questionnaire, followed by the RPMT, then by the WMT, then by the PPVT-III, and finally by the text-level task. In the case of high AQ individuals, these tasks were preceded by module 4 of the ADOS-2. The samples’ demographic, autistic, and cognitive profiles are detailed in Table 1.

**Table 1:** Groups’ demographic, autistic, and cognitive characterization.

	high AQ (*n* = 18)	low AQ (*n* = 18)	Statistics	*p* value
Demographic profile				
Sex (F:M)	9:9	7:11	*Χ*^2^_(1)_ = 0.11^a^	0.737
Years of age	22.72 (6.43)	19.94 (3.19)	*F*_(1,34)_ = 2.69^b^	0.110
Years of education	14.29 (1.78)	13.87 (1.46)	*F*_(1,34)_ = 1.35^b^	0.312
Autistic profile				
AQ	31.88 (3.61)	14.44 (1.62)	*F*_(1,34)_ = 349.78^b^	**< 0.001**
ADOS-2: Total score	9.94 (5.12)	–	–	–
ADOS-2: RSI subscore	6.05 (3.40)	–	–	–
ADOS-2: SBRI subscore	2.11(2.4)	–	–	–
ADOS-2: Comm subscore	3.88 (1.96)	–	–	–
Cognitive profile				
Mnesic skills (WMT)	18.05 (5.16)	21.05 (4.09)	*F*_(1,34)_ = 4.21^b^	**0.047**
Vocabulary (PPVT-III)	170.72 (10.95)	170.61 (6.06)	*F*_(1,34)_ = 0.001^b^	0.970
Non-verbal intelligence (RPMT)	111.61 (12.80)	111.16 (7.09)	*F*_(1,34)_ = 0.01^b^	0.898

(a) *p* values calculated with chi-squared test; (b) *p* values calculated with one way ANOVA. *RSI* reciprocal social interaction, *SBRI* stereotyped behaviors and restricted interests, *Comm* communication, *WMT* working memory task, *PPVT-III* Peabody Picture Vocabulary Test III, *RPMT* Raven’s Progressive Matrices Test.

All participants read and signed an informed consent form before beginning the study. The protocol was carried out in accordance with the principles of the Declaration of Helsinki and was approved by the Ethical Research Committees of Universidad de La Laguna.

### Discourse-level task

#### Naturalistic texts

All participants listened to two stories used in previous social concept research: an ST (highlighting interpersonal events) and an nST (narrating the activities of a single individual)^24^. The social/non-social contrast was manifested in the verbs and circumstantial adjuncts of each text. Most of these units in the ST referred to social interactions between two people (e.g., greeting someone kindly), whereas all verbs and circumstances in the nST lacked socio-interactive associations, as they described the actions of an unaccompanied character on various objects (e.g., preparing breakfast).

Both stories were composed through a systematic text-construction protocol^36–39, 46, 47^. First, 22 grammatical patterns were created and pseudo-randomly distributed for each text, each filled with strategic lexical items. For example, the pattern “Compound sentence: clause 1 [complement + empty subject + verb + complement] + conjunction + clause 2 [empty subject + verb + complement]” was filled as *Immediately, he went over to Juan and earnestly asked for a favor* for the ST, and as *Afterwards, he would read a book and listen to classical music on the balcony* for the nST. Both texts were matched for (1) character count; (2) overall and specific word-type counts; (3) mean content-word frequency, familiarity, syllabic length, number of letters, propositional density, and imageability; (4) sentence and sentence-type counts; and (5) a readability measure (Szigriszt-Pazos Index) and its associated readability rating (Inflesz scale). Moreover, the texts were matched for grammatical correctness, coherence, and comprehensibility (as judged by 20 raters on a scale from 1 through 5), as well as emotional content (positive, negative or neutral) and arousal level (intensity of the chosen emotion, from 1 through 5, as established by 14 raters). All sentences communicated mostly literal meanings and contained no jargon. See statistical details in Table 2. For full transcriptions and approximate English translations, see Supplementary material , Naturalistic texts. Stories were audio-recorded by a male native speaker of Canarian Spanish (the participants’ regional dialect), at a smooth pace, in .mp3 stereo format. Each narration lasted roughly 100 s.

**Table 2:** Linguistic features of the texts.

	Social text	Non-social text	Statistic	*P *value*
Characters^a^	959	949	*χ*^*2*^_(1)_ = 0	1
Words	199	199	*χ*^*2*^_(1)_ = 0	1
Nouns	43	40	*χ*^*2*^_(1)_ = 0.061	0.80
**Verbs**	**32**	**32**	*χ*^*2*^_(1)_ = 0	1
**Circumstantial adjuncts**	**28**	**30**	*χ*^*2*^_(1)_ = 0.020	0.89
**Social verbs**	**24**	**0**	*χ*^*2*^_(1)_ = 23.456	**< 0.001**
**Non-social verbs**	**8**	**32**	*χ*^*2*^_(1)_ = 14.703	**< 0.001**
**Social circumstantial adjuncts**	**15**	**0**	*χ*^*2*^_(1)_ = 13.578	**< 0.001**
**Non-social circumstantial adjuncts**	**13**	**30**	*χ*^*2*^_(1)_ = 6.675	**< 0.001**
Content word frequency^b^	1.7	1.81	*t*_(204)_ = 0.6666	0.50
Content word familiarity^b^	6.27	6.27	*t*_(204)_ = 1.4538	0.15
Content word imageability^c^	4.81	5.04	*t*_(204)_ = 0.2399	0.81
Content word syllabic length^c^	2.63	2.42	*t*_(204)_ = 1.2620	0.21
Content word orthographic length^c^	6.37	5.85	*t*_(204)_ = 1.1447	0.25
Sentences	22	23	*χ*^*2*^_(1)_ = 0	1
Minor sentences	3	4	*χ*^*2*^_(1)_ = 0.121	0.73
Simple sentences	8	8	*χ*^*2*^_(1)_ = 0.012	0.91
Compound sentences	4	5	*χ*^*2*^_(1)_ = 0.089	0.76
Complex/complex-compound sentences	7	6	*χ*^*2*^_(1)_ = 0.009	0.92
Grammatical correctness	3.75	4.24	*t*_(19)_ = 1.7366	0.09
Coherence	3.7	4	*t*_(19)_ = 1.1292	0.26
Comprehensibility	4.24	4.38	*t*_(19)_ = 0.7151	0.48
Szigriszt-Pazos Index^d^	74.81	72.26	–	–
Inflezs scale rating^e^	Fairly easy	Fairly easy	–	–
Emotional valence-neutral	36.04	37.58	*t*_(13)_ = 0.3814	0.71
Emotional valence-positive	60.71	61.18	*t*_(13)_ = 0.1128	0.91
Emotional valence-negative	1.62	0.31	*t*_(13)_ = 1.1888	0.25
Arousal-positive	3.18	2.47	*t*_(13)_ = 1.4235	0.18
Arousal-negative	0	0	*t*_(13)_ = 1.1329	0.34
Motor content^f^	2.55	3.01	*t*_(1)_ = − 1.74	0.04
Propositional density^g^	0.119	0.119	–	–

Significant values are in [bold].

(a) Character count was performed without counting spaces; (b) data was extracted from the LEXESP database, through B-Pal^48^; (c) data extracted from B-Pal^48^; (d) formula applied as described in Szigriszt Pazos^49^; (e) formula applied as described in Barrio-Cantalejo^50^; (f) data extracted from San Miguel Abella and González-Nosti^51^; (g) formula applied as described in Brown^52^; the asterisk (*) denotes alpha level set at. 0.5.

#### Comprehension questionnaires

Following each narration, participants completed a 16-item multiple-choice questionnaire featuring wh-questions^47^. Half the questions pointed to verb-related information, denoting the characters’ activities, and were mostly structured as *What did [a character] do when…?* The other half aimed at circumstances, realized by adverbial or prepositional phrases pointing to locative, causal, temporal, or social information signalled by *Where*, *Why*, *When* or *How*. In the ST questionnaire, all verb-related and circumstantial questions targeted social interactions (e.g., *How did Juan react to Albert’s payment offer? He rejected it*; *How did Albert insist? Kindly*). Conversely, in the nST questionnaire, all verb-related and circumstantial questions targeted non-social information (*What did Luis do with the TV? He turned it on*; *Where was the clock? On the nightstand*).

Questions were presented following the stories’ sequence of events, alternating between verb-related and circumstantial items. Successive questions were independent from each other. Each question featured five options: a correct response, three subtly incorrect options, and an ‘I don't remember’ option. Sequencing of options was randomized across questions, except for ‘I don’t remember’, which always appeared last. Correct responses were given one point; the others were given zero points. Each questionnaire had a maximum score of 16 points expressed as a percentage of correct answers for analysis.

### Procedure

Participants were instructed to close their eyes and listen carefully to the recorded texts through professional, high-definition headphones (Fig. 1B). At the beginning of the task, a different narrative was administered for familiarization purposes. It consisted in one text with the same length and structure as the ones in the experiment, followed by three sample questions on the computer screen. After this practice, participants listened to the ST and nST. Each text was played only once. Texts were counterbalanced across participants. Following each narration, its corresponding questionnaire was presented with its options. Participants were instructed to choose the correct answer as quickly as possible, using predefined keyboard keys. Selected options were automatically saved. The experiment ran in e-prime.

### Behavioral data analysis

First, we carried out a cross-textual analysis via a 2 × 2 mixed-effects ANOVA, with a between-subject factor ‘group’ (high AQ and low AQ) and a within-subject factor ‘text’. Then, given the mismatch in motor content (see Table 2), and as in previous works employing this discourse-level paradigm, we implemented a within-text analysis, comparing the performance between groups for each text separately^9, 38, 46^ via one-way ANOVAs, with ‘group’ as the categorical factor. We thus favored comparability with previous studies while circumventing confounds (fine-grained aspects not controlled between texts) and unduly stringent analyses. Also, to determine whether potential text retrieval outcomes were related to non-verbal intelligence, working memory, or vocabulary skills, results from the naturalistic text task were reanalyzed via ANCOVAs, covarying for the total scores of the RPMT, WMT, and PPVT-III, as in previous works^37^. Alpha levels were set at *p* < 0.05. Effect sizes were calculated via partial eta squared (η^2^) for ANOVAs and Cohen’s *d* for pairwise comparisons^53^.

To gauge the importance of social concept processing for discriminating individuals in each group, we performed linear discriminant analyses (LDAs). This method identifies the linear combination of a set of covariates that maximizes between-group differences while minimizing within-group differences^54^. We ran two models, one for the ST with four predictors (ST, RPMT, WMT, and PPVT-III scores) and one for the nST with four predictors (nST, RPMT, WMT, and PPVT-III scores). Performance estimates were corrected in a data-driven approach via leave-one-out cross-validation^55^. To determine which covariates best differentiated between high AQ and low AQ individuals in each LDA, we implemented a stepwise forward variable selection using the Wilk’s Lambda criterion. LDA classification results are reported through confusion matrices and receiver-operating characteristic (ROC) curves. All analyses were performed on R 4.1.1^56^.

Finally, to examine whether social concept outcomes were associated with autistic traits in the high AQ group, we performed Pearson’s partial correlations between performance on each text and four measures from the ADOS-2: the instrument’s total score as well as the ‘reciprocal social interaction’, ‘communication’, and ‘stereotyped behaviours and restricted interests’ subscales. These analyses were covaried by WMT scores, given that working memory was impaired in the high AQ group and has been shown to correlate with social cognition outcomes^57, 58^.

## Results

### Cross-textual analysis

The cross-textual analysis revealed a significant main effect of text (*F*_(1,34)_ = 15.71, *p* < 0.001, *η*^2^ = 0.11), with significantly higher scores for the ST (*M* = 66, SD = 17) than for the nST (*M* = 77.4, SD = 17). This effect remained significant even after controlling for RPMT (*F*_(1,33)_ = 14.71, *p* < 0.001), WMT (*F*_(1,33)_ = 16.63, *p* < 0.001), and PPVT-III (*F*_(1,33)_ = 14.63, *p* < 0.001) results. Additionally, the main effect of group approached significance (*F*_(1,34)_ = 3.59, *p* = 0.07, *η*^*2*^ = 0.07), with higher scores for the low AQ group (*M* = 76.2, *SD* = 15.6) compared to the high AQ group (*M* = 67.2, *SD* = 19.2). This trend remained after accounting for RPMT (*F*_(1,33)_ = 3.57, *p* = 0.07), WMT (*F*_(1,33)_ = 3.53, *p* = 0.07), and PPVT-III (*F*_(1,33)_ = 3.72, *p* = 0.06) results. Finally, the interaction between text and group was not significant (*F*_(1,34)_ = 0.92, *p* = 0.34, *η*^*2*^ = 0.007).

### Within-text analyses

ST scores were significantly lower for the high AQ group (*M* = 71, *SD* = 20) than for the low AQ group (*M* = 83, *SD* = 11) (*F*_(1,34)_ = 4.73, *p* = 0.037, *d* = 0.74) (Fig. 1C, left inset). This effect remained significant after covarying for RPMT (*F*_(1,33)_ = 4.60, *p* = 0.039), WMT (*F*_(1,33)_ = 4.86, *p* = 0.035), and PPVT-III (*F*_(1,33)_ = 4.67, *p* = 0.038) results. Conversely, nST scores did not differ significantly between groups (high AQ: *M* = 63, *SD* = 18; low AQ: *M* = 69, *SD* = 16; *F*_(1,34)_ = 1.19, *p* = 0.282, *d* = 0.35) (Fig. 1C, right inset). This result remained non-significant after covariation with RPMT (*F*_(1,33)_ = 1.16, *p* = 0.288), WMT (*F*_(1,33)_ = 1.24, *p* = 0.273), and PPVT-III (*F*_(1,33)_ = 1.16, *p* = 0.289) outcomes.

### Subject-level discrimination

The first LDA model showed that ST score was the only variable classifying between persons in the high AQ and the low AQ groups (Wilkis’s λ = 0.87, *F*_(1,34)_ = 4.73, *p* = 0.036). This model successfully classified 72% of participants (72% of high AQ and 72% of low AQ individuals, 95% CI 0.54–0.85, *p* = 0.005, Cohen’s Kappa = 0.44) (Fig. 1D, left inset), with an area under the ROC curve (AUC) of 64.20 (95% CI 0.45–0.84). Conversely, the second LDA model, including nST scores, yielded non-significant results. The variable that most contributed to group classification was WMT score (Wilkis's λ = 0.89, *F*_(1,34)_ = 4.21, *p* = 0.047). This model with nST scores only classified 58% of the participants (67% of high AQ and 50% of low AQ individuals, 95% CI 0.40–0.75, *p* = 0.203, Cohen’s Kappa = 0.16) (Fig. 1E, left inset), with an AUC of 53.09 (95% CI 0.34–0.74).

### Correlations between discourse-level scores and autism measures

Correlations between discourse-level scores and ADOS-2 outcomes in the high AQ group also differed between texts. Upon covarying for WMT results, ST outcomes were negatively correlated with the instrument’s total score (*r* ​ = − 0.51, *p* ​ = ​0.039) as well as the ‘reciprocal social interaction’ subscore (*r* ​ = ​− 0.55, *p* ​ = ​0.026), with the ‘stereotyped behaviors and restricted interests’ subscore (*r* = − 0.55, *p* = 0.02), but not with the ‘communication’ subscore (*r* = − 0.38, *p* = 0.13) (Fig. 1F). Contrariwise, nST scores were not significantly associated with any such measures (total score *r* ​ = ​− 0.37, *p* ​ = ​0.154; ‘reciprocal social interaction’: *r* ​ = ​− 0.40, *p* ​ = ​0.122; ‘stereotyped behaviors and restricted interests’: *r* = − 0.33, *p* = 0.21; ‘communication’: *r* = − 0.27, *p* = 0.30) (Fig. 1G).

## Discussion

This is the first study to examine (text-level) social concept comprehension in persons with and without autistic-like traits. Unlike nST scores, ST scores were significantly lower in the high than in the low AQ group, and this result was uninfluenced by individual variability in non-verbal intelligence, working memory, and vocabulary skills. Moreover, performance on the ST (but not on the nST) robustly classified subjects as high AQ or low AQ, and it was negatively associated with total ADOS-2 score and relevant subscales. Below we discuss our findings, addressing their theoretical implications.

Our key finding is that high AQ participants were outperformed by their low AQ counterparts on the ST but not on the nST. This aligns with research revealing impaired processing of social semantic content in persons with ASD^59^ and selective ST deficits in neurodegenerative patients with primary socio-affective impairments^24^. Suggestively, social (relative to emotional) concepts distinctly recruit fronto-temporo-parietal regions (e.g., prefrontal cortex, middle temporal gyrus, temporo-parietal junction)^60^ that present anatomo-functional alterations in ASD^61–64^, especially during social cognition tasks^65, 66^. Our results support and extend these findings, suggesting that social concept skills may be partly driven by situated interpersonal experience.

The ST difficulties of the high AQ group emerged independently of non-verbal intelligence, working memory, and vocabulary level. Previous studies on ASD and high AQ samples suggest that these domains may influence performance on other socio-cognitive domains^67^, such as theory of mind^68^, emotion recognition^69^, and socializing^70^. However, no such influences have been detected in other studies^32, 33^, suggesting that only certain socio-cognitive domains, or certain socio-cognitive tasks, are influenced by such general skills. In this sense, our results indicate that selective text-level social concept deficits in high AQ persons may not be secondary to broader cognitive dysfunctions, but rather represent a *sui generis*, category-specific semantic deficit. This further suggests that social concept processing may be grounded in interpersonal experience, beyond the latter’s effects on other cognitive functions.

Interestingly, the cross-textual analysis revealed significantly lower scores on the nST than on the ST. This discrepancy could be influenced by the nST’s greater motoric content—namely, the level of bodily movement implied by verbs^51^. Indeed, stimuli with high motor content prove more cognitively challenging than those with low motor content^71, 72^. The interaction between group and text, however, was not significant. Such an effect, we surmise, may have been abolished by the greater demands of the nST (conceivably, a less demanding control text could have elicited better outcomes in both groups, increasing the performance difference relative to the ST). While this remains speculative, such a null result further emphasizes the importance of employing within-text analyses to elucidate condition-specific differences between groups^9, 38, 46^.

In this sense, LDA results showed that ST (unlike nST) scores discriminated *individual* high AQ participants from low AQ participants. ST scores emerged as the best classifier between groups, with an accuracy of 72% (and perfect balance between both groups). This variable even outweighed WMT scores—which is notable given the systematicity of working memory deficits in persons with high AQ^73^ and ASD^74^. Thus, group-level results were not dependent on a few low-scoring participants. Indeed, social semantic outcomes in natural speech also constitute the most accurate variable for classifying persons with and without ASD^75^. In people with reduced social experience, then, ST comprehension difficulties seem inter-individually consistent and more discriminatory than other cognitive deficits.

Moreover, ST outcomes in the high AQ group were negatively correlated with ADOS-2 scores (adjusted for WMT scores). This reinforces the claim that social concept processing hinges on interpersonal experience. Indeed, in ASD research, ADOS-2 scores have been shown to correlate with deficits on other social cognition tasks^31, 76, 77^. Notably, subscale analyses revealed that ST outcomes were correlated with the ‘reciprocal social interaction’ (ability to engage in exchanges with one or more people) and the ‘stereotyped behaviors and restricted interests’ subscores (conducts repeatedly in an exact way, often involving individual actions). Scores on these subscales have been associated with domains like emotion recognition^78, 79^ and theory of mind^80, 81^, attesting to their relevance to socio-cognitive skills at large. Importantly, no correlations with ADOS-2 scores emerged for the nST, even though it proved more demanding than the ST. This further suggests that socio-cognitive variability across high AQ participants was not related to difficulties with linguistic materials at large, but rather confined to texts conveying social information. Thus, though strictly correlational, our results lend additional support to our hypothesis, as they indicate that the poorer the engagement with others, the lower the capacity to grasp social information.

Taken together, our findings indicate that the ability to process social concepts is related to situated gregarious experience. This evidence supports the view that interpersonal exchanges are distinctly needed to acquire and use diverse abstract concepts, including social ones^5^. From a situated cognition perspective, concepts become consolidated by virtue of experiencing the events or referents they denote^13–15^, so that their ulterior activation would involve accessing relevant memory traces^13, 16^. Such traces, we propose, would be suboptimally entrenched in hAQ individuals given their reduced social experience. In fact, social isolation has been linked to abnormal activation of prefrontal, superior temporal, and temporo-parietal circuits^82, 83^ specifically recruited during social concept processing^60^. Accordingly, we speculate that atypicalities in such regions due to low social engagement would render social concepts harder to access or even construe (a conjecture that should be examined in future studies).

Importantly, however, we cannot ascertain whether present results are specific to social concepts or general to abstract concepts at large. Indeed, prominent accounts propose that sustained interpersonal experience is critical for grounding all types of abstract concepts, including emotional, philosophical, and spatiotemporal ones^5^. Unfortunately, the nST (our control condition) involved action events rather than another subcategory of abstract concepts, such as emotional concepts. Still, the ST and the nST were controlled for emotional valence and arousal, partly ruling out some such confounds. Relatedly, social concepts have been shown to make up a differentiated lexico-semantic space^6^ and to engage socio-cognitive regions significantly more than emotional concepts^60^. By the same token, our study warrants the view that social concepts might be especially (though not exclusively) dependent on interpersonal experience.

Finally, note that our findings stemmed from naturalistic narratives. Most social concept investigations have relied on randomized sequences of isolated words or sentences^7, 17–20^, while discourse-level studies on ASD have targeted syntactic^84^ and macro-structural^85^ aspects. Despite their major contributions, such approaches fail to capture social concepts in context-rich settings. The detection of category-specific difficulties in this study offers important support for such links, as contextual cues in natural texts may facilitate performance by priming or favoring maintenance of relevant information^86^. In this sense, our study meets the pressing call for more ecological insights on language processing^37, 41, 87^.

This study is not without limitations. First, our groups were relatively small, calling for replications with more participants. Second, the number of testing items was limited. Although our task doubles the number of trials in previous studies^39, 46^, future adaptations should increase this figure. Third, our protocol does not capture the relative contributions of mentalistic, empathic, or emotional abilities to the observed outcomes. Accordingly, present results may warrant alternative or complementary explanations, given that STs likely engaged these domains, which are affected in high AQ individuals^28–30^. Future works should examine or rule out such factors in this population by leveraging classical fine-grained tasks (e.g., synonym judgment, semantic feature verification)^34^ or by incorporating relevant socio-cognitive measures based on non-verbal stimuli—e.g., facial emotion recognition^88^ or picture-based empathy measures^89^. Fourth, future studies on high AQ individuals should leverage more tightly controlled social and non-social stimuli to enable stringent tests of group-by-condition interactions. Finally, we call for new studies to expand social concept research by involving persons with actual ASD diagnoses, including offline or online neural measures, and performing interventions to enhance or manipulate social experiences (e.g., via virtual reality) and examine their direct impact on social concept processing.

In conclusion, we showed that persons with reduced interpersonal experience presented difficulties with grasping text-level social concepts, and that such difficulties correlated with validated measures of social interaction. This evidence supports situated views of semantic processing, affording new insights on (a particular type of) abstract concepts. Future applications of our approach could promote useful breakthroughs to understand the links between (outward) social events and their (inner) cognitive construal.

### Supplementary Information

Supplementary Information.

## Data Availability

All experimental data, as well as the scripts used for their collection and analysis, are available via the Open Science Framework at http://bit.ly/3js012x.
