# Supplementary material for: Impaired social concept processing in persons with autistic-like traits
Source: Sci Rep. 2023 Sep 21;13:15709. doi: 10.1038/s41598-023-42889-2 (PMC10514259; doi:10.1038/s41598-023-42889-2)
Supplement: Supplementary file 1 — Supplementary Information. [file 41598_2023_42889_MOESM1_ESM.docx]

**Supplementary materials**

**Power estimation**

To determine the sample size required for our one way ANOVA analyses, we ran a power estimation on *R* with the pwr library. We established an alpha level of 0.05, an effect size of η^2^ = 0.2, and a power of 0.8, based on effect sizes from previous study on persons with and without autistic-like traits [^1^](#_ENREF_1). The final sample size of our study (with the smallest group featuring 18 participants) reaches a power of .83.

**Cognitive assessments**

**General intelligence.** General intelligence was assessed with Raven’s Progressive Matrices Test, which measures deductive ability (ability to make sense of complex situations) by having participants select the missing piece in a figure. The test is suitable for testing all ages from 5 to 90. The booklet comprises five sets (A to E) of 12 items each (e.g., A1 through A12), with items within each set becoming increasingly difficult, but then reverting to easy items at the beginning of the next set. This cyclical presentation provides training in the method of thinking required to solve the problems. All items are printed in black ink on a white background. The maximum score is 135 [^2^](#_ENREF_2).

**Mnesic skills.** Auditory verbal WM was assessed via the Spanish adaptation of a validated listening span test [^3^](#_ENREF_3). Participants were instructed to complete the missing final words of unrelated sentences. The missing words were concrete disyllabic nouns [e.g., “*In summer it is very*____” (“*En verano hace mucho*___”)]. Then, participants had to repeat all the selected words –in this case, *hot* (*calor*). The task increased the number of items in each level, starting with two and finishing with five items. Task administration was stopped when the participant failed to retrieve at least one item at a given level. Correct and incorrect answers received one and zero points, respectively. To calculate the absolute span score, the number of words recalled on perfectly recalled trials was summed. In perfectly recalled trials, participants were not penalized for recalling the words in an incorrect order. The highest possible absolute span score was 42.

**Receptive vocabulary.** Receptive vocabulary knowledge was measured with the validated Spanish version of the Peabody Picture Vocabulary Test-III [^4^](#_ENREF_4), with a test-retest reliability of .93 and an internal consistency between .89 and .97. The test consists of 16 levels with 12 trials containing four pictures each. For adult people, only levels from 12 to 16 are used. In each case, the examiner said a word and participants had to select which of the pictures represented its meaning. Correct responses received one point and incorrect ones, zero points. The task was finished when the participant had 8 or more errors in the same level (having each level a total of 12 of trials). The score was calculated as the number of the highest trial reached minus the number of errors committed. The maximum score is 192.

**Naturalistic texts**

**Supplementary Table 1.** Full transcriptions and approximate translations of the texts.

| **Spanish originals** | |
| --- | --- |
| **Social text**  ¡Al fin llegaron las vacaciones! Alberto había solicitado dos semanas de descanso y su jefe se las dio. Cogió las maletas y salió de la casa. Juan, su vecino jardinero, lo saludó amistosamente, con una sonrisa. Le deseó un buen viaje. Al hablar con Juan, Alberto recordó su jardín. ¡Sus hermosas flores! ¿Quién las regaría? El taxi en la esquina, a segundos de la casa. ¡Cuánta emoción! Inmediatamente se reunió con Juan y le pidió encarecidamente el favor. Le explicó su situación y le ofreció dinero para que cumpliera con su pedido. ¡Era la persona ideal para ayudarlo! Ya en el taxi, mientras esperaba la respuesta de su vecino, Alberto le indicaba el recorrido al conductor. Juan aceptó con gusto la tarea que Alberto le encargó y rechazó cualquier tipo de pago. Con amabilidad, Alberto insistió y le dio el dinero. Juan se lo devolvió gentilmente. Había mostrado un gesto de amistad. Alberto se lo retribuiría. Luego, a través de la ventana del taxi, le entregó a Juan las llaves para entrar a su casa. Durante todo el viaje, Alberto agradeció la ayuda de su vecino Juan. Como devolución, al reencontrarse con él, le regalaría una de sus flores. | **Non-social text**  Luis abrió los ojos y miró el reloj de la mesita. Había dormido profundamente durante nueve horas. ¡Al fin era fin de semana! Aún en pijama y con las sandalias puestas, fue a la cocina y calentó agua para hacerse un café. ¡Iba a preparar un rico y completo desayuno! Mientras tostaba rodajas de pan, servía cereales en un plato. ¡Qué relajación! ¡Le encantaba el olor del café recién hecho! Como todos los días, para tomarlo, usó su taza preferida. Luego, durante el desayuno, Luis planificaba qué hacer a lo largo de su día. Primero vería su película favorita. Después leería un libro y escucharía música clásica en el balcón. Casi el mediodía. ¡Qué bueno! ¡Toda la tarde entera por delante! Prendió el televisor y puso la película con muchas ganas. Finalizada la película, leyó varias páginas del libro en el sofá. Más tarde, mientras escuchaba música en el tocadiscos, salió al balcón. Allí, observó el paisaje y tomó aire fresco. Era un atardecer hermoso. Se sentía lleno de energía. Entonces, fue al parque más verde de toda la ciudad. Hizo deporte aeróbico durante una hora antes de recostarse y relajarse en el pasto recién cortado. |
| **Approximate English translations** | |
| **Social text**  Vacation time has finally arrived! Albert had requested to take two weeks off, which his boss approved. He grabbed his bags and left the house. John, his gardener neighbor, greeted him with a friendly smile. He wished him a pleasant trip. Talking to John, Albert pondered on his garden. His beautiful flowers! Who would water them? The taxi was at the corner, seconds away from the house. How exciting! He immediately joined John and emphatically asked him the favor. He explained his situation and offered him money to fulfill his request. He was the ideal person to help him! Inside the taxi, while waiting for his neighbor’s response, Albert gave the driver directions. John gladly accepted the task Albert had entrusted him with and refused any type of payment. With kindness, Albert insisted and handed him the money. John gave it back politely. He had shown a gesture of friendship. Albert would give him something in return. Later, through the taxi’s window, he passed John the keys to his house. During his trip, Albert was thankful for the help of his neighbor John. As a token of gratitude, when meeting him again, he would gift him one of his flowers. | **Non-social text**  Jack opened his eyes and looked at the clock on his nightstand. He had been sound asleep for nine hours. The weekend had finally arrived! Still in his pajamas and wearing his sandals, he went into the kitchen and heated up water to make some coffee. He was about to prepare a tasty and hearty breakfast! As the sliced bread was being toasted, he served cereal on a plate. It was so relaxing! He loved the smell of freshly brewed coffee! As he did every day, he used his favorite mug to drink it from. Later, during breakfast, Jack planned what to do during the day. He would first watch his favorite movie. Then, he would read a book and listen to classical music on the balcony. It was almost noon. That was great! The entire evening was still ahead! He turned the TV on and played the movie with excitement. Once the movie was over, he read several pages of his book while sitting on the couch. Later, as he was listening to music on the turntable, he went out into the balcony. There, he looked at the landscape and enjoyed the fresh air. It was a beautiful sunset. He felt full of energy. At this point, he visited the greenest park in the city. He did aerobics for an hour before laying and relaxing on the recently mowed grass. |
| Note: The English translations above are simply communicative renditions for the benefit of readers who cannot read in Spanish. These English texts do not replicate the strict control of variables present in the Spanish originals. | |

**References**

1 Padrón, I. *et al.* Multisession anodal tDCS on the right Temporo-Parietal Junction improves mentalizing processes in adults with autistic traits. *Brain sciences* **12**, 30 (2021).

2 Raven, J. in *Handbook of nonverbal assessment* 223-237 (Springer, 2003).

3 Rodrigo, M. J., Padrón, I., De Vega, M. & Ferstl, E. C. Adolescents’ risky decision-making activates neural networks related to social cognition and cognitive control processes. *Frontiers in human neuroscience* **8**, 60 (2014).

4 Dunn, L. & Dunn, L. Examiner’s Manual for the PPVT-III. Form IIIA and IIIB. *Technical report* (1997).
